# Critical role of aldehydes in cigarette smoke-induced acute airway inflammation

**DOI:** 10.1186/1465-9921-14-45

**Published:** 2013-04-17

**Authors:** Marco van der Toorn, Dirk-Jan Slebos, Harold G de Bruin, Renee Gras, Delaram Rezayat, Lucie Jorge, Koen Sandra, Antoon JM van Oosterhout

**Affiliations:** 1 Department of Pathology & Medical Biology, Lab. Allergology & Pulmonary Diseases, Hanzeplein 1, Groningen, GZ, 9713, The Netherlands; 2 GRIAC Research Institute, Groningen, The Netherlands; 3 Department of Laboratory Medicine, Groningen, The Netherlands; 4 Department of Pulmonary Diseases, University Medical Center Groningen, University of Groningen, PO Box 30001, Groningen, RB, 9700, The Netherlands; 5 Metablys, Research Institute for Chromatography, President Kennedypark 26, Kortrijk, 8500, Belgium

**Keywords:** Cigarette smoke, Aldehydes, Mouse model, Airway inflammation, COPD

## Abstract

**Background:**

Cigarette smoking (CS) is the most important risk factor for COPD, which is associated with neutrophilic airway inflammation. We hypothesize, that highly reactive aldehydes are critical for CS-induced neutrophilic airway inflammation.

**Methods:**

BALB/c mice were exposed to CS, water filtered CS (WF-CS) or air for 5 days. Levels of total particulate matter (TPM) and aldehydes in CS and WF-CS were measured. Six hours after the last exposure, inflammatory cells and cytokine levels were measured in lung tissue and bronchoalveolar lavage fluid (BALF). Furthermore, Beas-2b bronchial epithelial cells were exposed to CS extract (CSE) or WF-CS extract (WF-CSE) in the absence or presence of the aldehyde acrolein and IL-8 production was measured after 24 hrs.

**Results:**

Compared to CS, in WF-CS strongly decreased (CS; 271.1 ± 41.5 μM, WF-CS; 58.5 ± 8.2 μM) levels of aldehydes were present whereas levels of TPM were only slightly reduced (CS; 20.78 ± 0.59 mg, WF-CS; 16.38 ± 0.36 mg). The numbers of mononuclear cells in BALF (p<0.01) and lung tissue (p<0.01) were significantly increased in the CS- and WF-CS-exposed mice compared to air control mice. Interestingly, the numbers of neutrophils (p<0.001) in BALF and neutrophils and eosinophils (p<0.05) in lung tissue were significantly increased in the CS-exposed but not in WF-CS-exposed mice as compared to air control mice. Levels of the neutrophil and eosinophil chemoattractants KC, MCP-1, MIP-1α and IL-5 were all significantly increased in lung tissue from CS-exposed mice compared to both WF-CS-exposed and air control mice. Interestingly, depletion of aldehydes in WF-CS extract significantly reduced IL-8 production in Beas-2b as compared to CSE, which could be restored by the aldehyde acrolein.

**Conclusion:**

Aldehydes present in CS play a critical role in inflammatory cytokine production and neutrophilic- but not mononuclear airway inflammation.

## Background

Smoking is the main causative factor for the development and progression of Chronic Obstructive Pulmonary Disease (COPD), a disease which is characterized by chronic airway inflammation and progressive airflow limitation [1,2]. The respiratory inflammatory infiltrate in COPD patients predominantly consists of inflammatory cells in the lung tissue and airways, of which neutrophils, macrophages and CD8+ T-cells are the most important. Airway epithelial cells are the first line of defense in the airways to cigarette smoke (CS) and are able to respond to CS with increased production of proinflammatory cytokines e.g. monocyte chemotactic protein-1 (MCP-1), macrophage inflammatory protein-1α (MIP-1α), granulocyte-macrophage colony stimulating factor (GM-CSF), IL-8 and IL-1ß [3,4]. These cytokines may play a role in the influx of inflammatory cells, in particular macrophages and neutrophilic granulocytes into the lung [5-7].

While the specific identity of compounds in CS that are responsible for this airway inflammation are still unknown, reactive oxygen species, reactive nitrogen species and free radicals are thought to play a key role in the induction of the inflammatory response present in COPD [8]. However, reactive components with unpaired valence shell electrons are not capable of diffusing through the plasma membranes and are rapidly neutralized by enzymatic antioxidants like superoxide dismutase, catalase, glutathione peroxidase and thioredoxin reductase or by nonenzymatic antioxidants glutathione and vitamine c all present in high concentrations in the epithelial lining fluid [9-11]. In addition to these short-lived reactive components, the gas phase of CS contains more stable oxidizing intermediates that have the potential to induce endogenous oxidative stress and inflammation, including aldehydes such as acetaldehyde, acrolein and crotonaldehyde [12]. In current smokers and patients with COPD, increased levels of aldehydes have been documented in exhaled breath condensate and saliva [13-15]. Furthermore, acrolein is involved in increased mucin production and regulation of lung matrix metalloproteinase 9 both associated with rapid decline in lung function and frequency of exacerbations in COPD patients [16]. We recently showed that acrolein as well as crotonaldehyde can irreversibly modify glutathione in human airway epithelial cells in vitro, rendering it unavailable for the maintenance of the cellular redox status [17]. Furthermore, aldehydes in CS are able to initiate release of IL-8 by human bronchial epithelial cells and enhance proinflammatory cytokines (e.g. TNF-α and IL-8) through activation of human macrophages [4,18]. Studies in human neutrophils showed that aldehydes are able to inhibit human neutrophil apoptosis, promoting longevity and thus contribute to neutrophilic accumulation [19]. All together these data suggest a critical role for aldehydes in modification of proteins leading to altered protein function and impaired regulation of redox-sensitive factors like NF-κB which may drive the CS-induced neutrophilic airway inflammation [20-22].

We therefore hypothesize that aldehydes present in CS play an important role in the production of proinflammatory cytokines and the subsequent recruitment of neutrophils into the airways. To study this hypothesis we first demonstrate that filtering of CS through water strongly decreases the levels of aldehydes, but has a minor effect on the concentration of other components in CS. We compared the effects of water filtered CS (WF-CS) with normal CS in an established mouse model of CS-induced neutrophilic airway inflammation.

## Methods

### Smoking conditions

Kentucky research reference cigarettes 3R4F (Tobacco Research Institute, University of Kentucky, Lexington, KY) were used for experiments. Different smoking conditions were made as described previously [10]. Briefly, just before the experiment, filters were cut from the cigarettes. Each cigarette was smoked in 5 minutes with a 10 mm butt remaining using a peristaltic pump (Watson Marlow 603S, Rotterdam, The Netherlands) [10]. CS was obtained by smoking cigarettes at a rate of 5 L/hr. WF-CS was obtained as described for CS, but the smoke was first passed through a receptacle of water (1L). For in vitro experiments, CS extract (CSE) was freshly prepared by bubbling two cigarettes through 25 ml of RPMI 1640 (Bio Whittaker, Verviers, Belgium), and this was regarded as 100% strength. WF-CS extract (WF-CSE) was produced as described for CSE, but the smoke was first passed through a receptacle of water (1L) before it was bubbled through RPMI 1640, and this was regarded as 100% strength.

### Quantification of total particulate matter in CS and WF-CS

CS total particulate matter (TPM) was collected from the mainstream smoke of CS and WF-CS by trapping on a Sterlitech GF-75 glass fiber filter disc (Sterlitech, Kent, USA). Increase in weight of filters was measured directly after collection of TPM.

### Quantitative determination of total aldehydes in CS and WF-CS

CS or WF-CS from 5 cigarettes was passed though 25 ml deionised water. Water samples were collected after each cigarette. Total concentration of aldehydes was measured with Amplite™ Colorimetric Aldehyde Quantitation Kit (Enzo Life Sciences, Antwerpen, Belgium) according to manufacturer’s instructions.

### Comparative analyses of CS and WF-CS using gas chromatography–mass spectrometry

Cigarettes were smoked using a Borgwaldt RGA System LM1 (Borgwaldt kc, Hamburg, Germany) in the ISO smoking regime. For each experiment, 5 puffs were made plus one cleaning puff. The filter of the cigarette was removed and tape was placed around the cigarette. The outlet of the smoking machine was connected to an impinger (Supelco, Bellefonte, USA). For CS an empty impinger was used and for WF-CS, 15 mL water (HPLC grade, Biosolve, Valkenswaard, NL) was placed in the impinger.

Thermal desorption combined with gas chromatography–mass spectrometry (GC-MS) was performed on a Gerstel TDS system combined with an Agilent 6890GC-5973MSD system (Agilent Technologies, Wilmington, DE, USA). The Tenax tubes were desorbed at 250°C. Volatile organic compounds are trapped in a cold trap at −100°C and injected in the GC. Separation was done on a 60 m × 0.25 mm ID × 1.4 μm HP-624 column (Agilent) by programming from 40°C to 250°C at 10°C/min (10 min hold). MS was operated in scan mode.

### Analysis of aldehydes present in CS and WF-CS

Cigarettes were smoked as described above. For aldehyde collection, the outlet of the impinger was connected to a LpDNPH S10 cartridge (Supelco). This adsorbent tube consists of octadecyl silica material impregnated with 2,4-dinitrophenylhydrazine (DNPH). Aldehydes and ketones are trapped and derivatized into the corresponding hydrazones. After collection, the derivatized solutes were eluted with 2 mL acetonitrile (HPLC grade, Biosolve), filtered (0.45 μm filter) and analyzed by liquid chromatography-ultraviolet spectrophotometry-mass spectrometry (LC-UV-MS). From the extracts, 5 μL was injected onto an Agilent Technologies 1290 Infinity UHPLC system (Agilent Technologies, Waldbronn, Germany), equipped with a diode array detector, hyphenated to an Agilent Technologies 6460 triple quadrupole mass spectrometer. Separation was done on a 2.1 mm i.d., 150 mm L, 1.8 μm d_p_ Zorbax SB C18 column (Agilent Technologies) using 10 mM ammoniumacetate (Biosolve) as mobile phase A and acetonitrile (Biosolve) as mobile phase B. Flow rate was 0.3 mL/min with a linear gradient from 10%B to 90%B during 10 min. Column temperature was maintained 40°C. UV detection was performed at 360 nm. The MS system was operated in MS2 scan mode and in selected ion monitoring (SIM) mode using Jetstream negative ionization. Typically [M-H]^-^ ions were detected. SIM traces were integrated in the MassHunter software package. Peak identity was confirmed using an aldehyde/ketone DNPH mix (Supelco).

### Animals

Specified pathogen-free female BALB\cByJ mice (8 weeks old) were obtained from Charles River (Lille, France). Mice were housed in individually ventilated cages with food and water ad libitum. Experiments were approved by the Institutional Animal Care and Use Committee of the University of Groningen (The Netherlands).

### Exposure of mice to CS, WF-CS and air

Mice were exposed to CS or WF-CS twice a day for five days, using respectively 1 and 3 cigarettes for the CS exposure on the first day and 5 cigarettes for every exposure on the subsequent days. Each cigarette was smoked in 5 minutes at a rate of 5 L/hr in a ratio with 60 L/hr air using whole body exposure. Gaseous-phase of CS or WF-CS was directly distributed inside 6-liter perspex boxes. As a control, mice were treated in an identical manner, exposing them to 60 L/hr air directly distributed inside 6-L perspex boxes with the same frequency and duration of the treatments as the CS-treated groups. On the last day a single CS, WF-CS or air control exposure was performed, and 6 hours thereafter bronchoalveolar lavage (BAL) was performed, and lungs were collected for further analysis as described below.

### Bronchoalveolar lavage and cell differentiation

Immediately after anesthesia and bleeding, the lungs of mice were lavaged 5 times through a tracheal cannula. The first BAL was performed with 1 ml PBS containing BSA (5%) and protease inhibitors (Complete mini, Roche Diagnostics, Penzberg, Germany). Cells were pelleted, and supernatants were stored at −80°C for cytokine measurements. Subsequently, lungs were lavaged with 4 ml PBS containing 1% BSA, and BAL cells were pooled and counted using a coulter counter (Z1™ Series, Beckman Coulter, Woerden, The Netherlands).

For differential cell counts, cytospin preparations were made using a cytocentrifuge (Shandon Life Science, Cheshire, UK), and cells were fixed and stained with Diff-Quick (Dade, Düdingen, Switzerland). Cells were identified and differentiated into mononuclear cells, eosinophils and neutrophils by standard morphology and staining characteristics. Per cytospin, 200 cells were counted and the absolute number of each cell type was calculated.

### Single cell suspension of lung tissue

The left lung lobes of the mice were transferred into a petri dish. Using a scalpel the lungs were cut in pieces. Lung pieces were then transferred to RPMI/BSA 1% + collagenase A (6.5 mg/ml) + DNAse I (0.1 mg/ml) and incubated at 37°C for 1 h. Using a 5 ml syringe plunger, the cells were forced through a 70 μm cell strainer (BD Bioscience, Alphen aan de Rijn, The Netherlands) placed on top of a 50 ml falcon tube (BD Biosciences, Alphen aan de Rijn, The Netherlands). Cells were washed with RPMI/BSA 1%, pelleted (300 g, 4°C, 5 min) and resuspended in lysis buffer for 5 minutes (0.15M NH_4_CL, 0.01 M KHCO_3_ and 0.1mM EDTA, pH 7.4) on ice. After incubation cells were washed with RPMI/BSA 1% and counted by a coulter counter. Cytospin preparations were made and cell differentials analyzed as described in the previous paragraph.

### Lung tissue homogenates

After BAL was performed, the right lung lobes of the mice were homogenized in 50 mM Tris–HCl buffer containing 150 mM NaCl, 0.002% Tween-20 and protease inhibitors (Complete Mini, Roche Diagnostics GmBH, Germany). Lung homogenates were made using a homogenizer (T10 Ultra-Turrax, IKA, Staufen, Germany) and subsequently they were centrifuged (12000 g, 4°C, 20 min) and supernatants were stored at −80°C until further analyses.

### Cytokines

Concentrations of MCP-1, GM-CSF, MIP-1α, IL-1β, IL-5, and KC in lung tissue homogenates and BALF were measured with a multiplex ELISA (Invitrogen, Bleiswijk, The Netherlands) according to the manufacturer’s instructions.

### In vitro *cell culture*

The human bronchial epithelial cell line (Beas-2b) was purchased from American Type Culture Collection (ATCC, Manassas, USA). Cells were grown in RPMI 1640 with 25 mM HEPES, L-Glutamine (Bio Whittaker, Verviers, Belgium) supplemented with 10% heat inactivated fetal calf serum (Bio Whittaker, Verviers, Belgium) and 1% Pen/Strep (Pen Strep, Invitrogen, Bleiswijk, The Netherlands). Cells were cultured in 25 cm^2^ culture flasks (Costar, Cambridge, UK) and 24 wells tissue cell culture plates (Costar, Cambridge, UK) at 37°C in an atmosphere of 5% CO_2_. Before the experiments cells were incubated overnight in serum free RPMI 1640 media.

### Stimulation of Beas-2b cells

Beas-2b cells were grown in 24 well tissue culture plates as described above. Cells were prestimulated for 30 minutes with control medium, acrolein (60, 90 and 120 μM), CSE (100%), WF-CSE (100%) or WF-CSE (100%) + acrolein (60, 90 and 120 μM). Thereafter, cells were washed twice and incubated for 24 h in culture medium alone. Next day, supernatants were collected centrifuged for 5 min at 600g, 4°C and stored at −80°C until further analyses.

### IL-8 ELISA

IL-8 levels were measured in supernatants of Beas-2b cells by sandwich ELISA using a human IL-8 ELISA kit (Sanquin, Amsterdam, The Netherlands) according to manufacturer’s instructions.

### Statistical analysis

**Figure 1:**
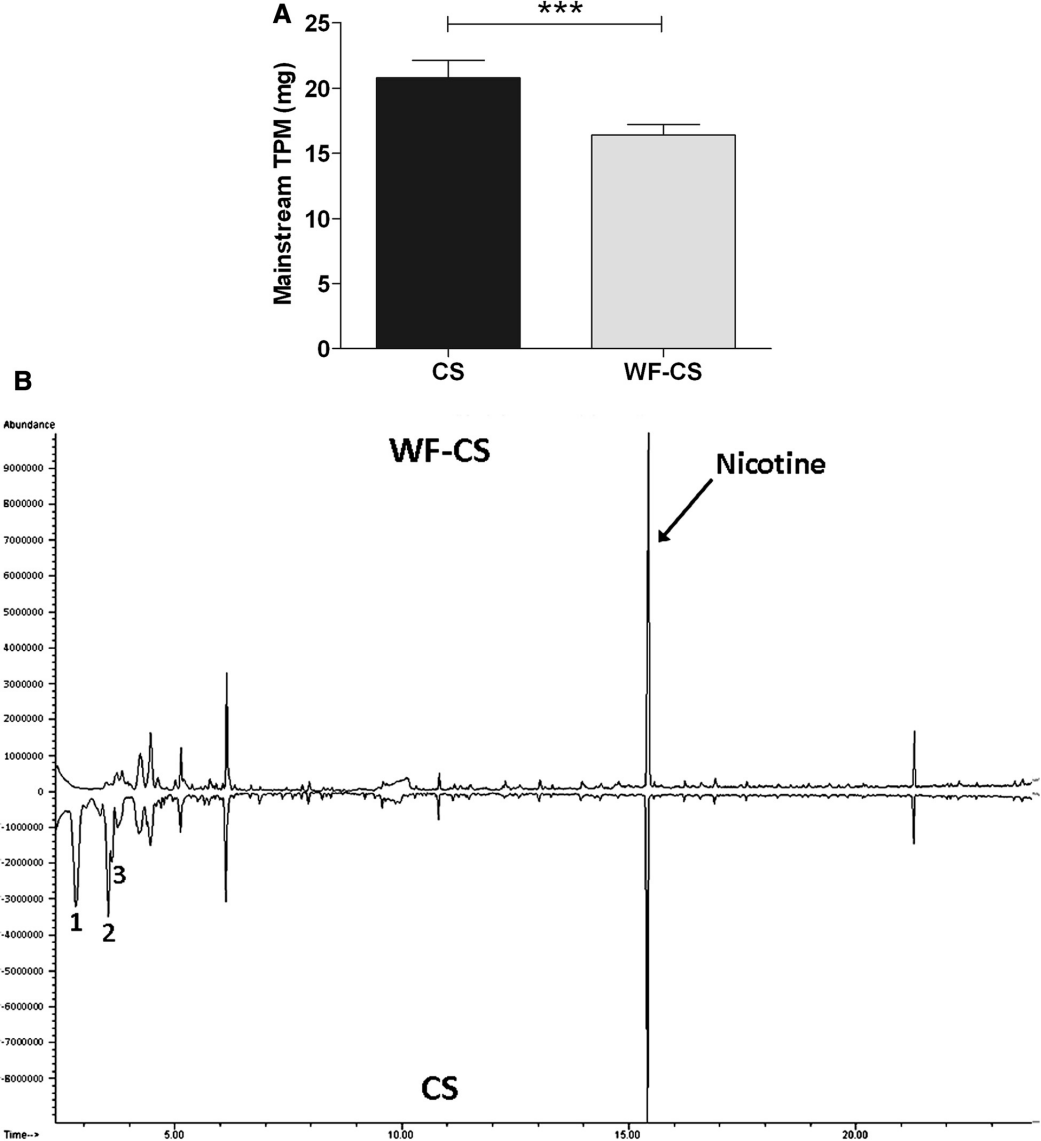
**Comparative analyses of total particulate matter (TPM) and volatiles in mainstream cigarette smoke (CS) and water-filtered cigarette smoke (WF-CS).** (**A**) Quantification of TPM in mainstream CS and WF-CS by trapping particles from 1 cigarette on glass fiber filters. Data are expressed as mean values ± SEM and are referred to 4 experiments. *** *P* < 0.0001 for comparison between CS and WF-CS group. (**B**) Comparative analysis of CS and WF-CS using GC-MS. Characterization of the peaks at time 2.8 (component 1), time 3.56 (component 2), time 3.64 (component 3) and time 15.4 (nicotine) was done by mass spectrometry.

**Figure 2:**
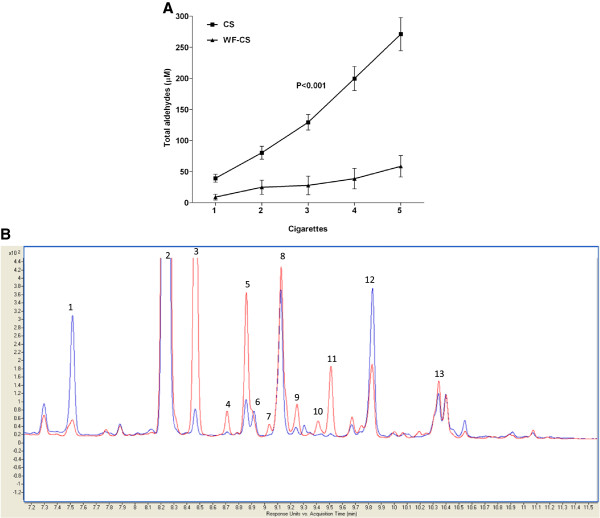
**Comparative smoke analysis of total aldehydes in cigarette smoke (CS) and water-filtered cigarette smoke (WF-CS).** (**A**) Quantification of total aldehydes in CS and WF-CS from 5 cigarettes. Data are expressed as mean values ± SEM and are referred to 4 experiments. *P* value given above the line represents the significance between CS and WF-CS. (**B**) Overlay of the HPLC-UV-chromatograms obtained for smoke analysis with the impinger filled with water (blue) and the impinger left empty (red). Peak annotations and fold change are reported in Table 1.

**Figure 3:**
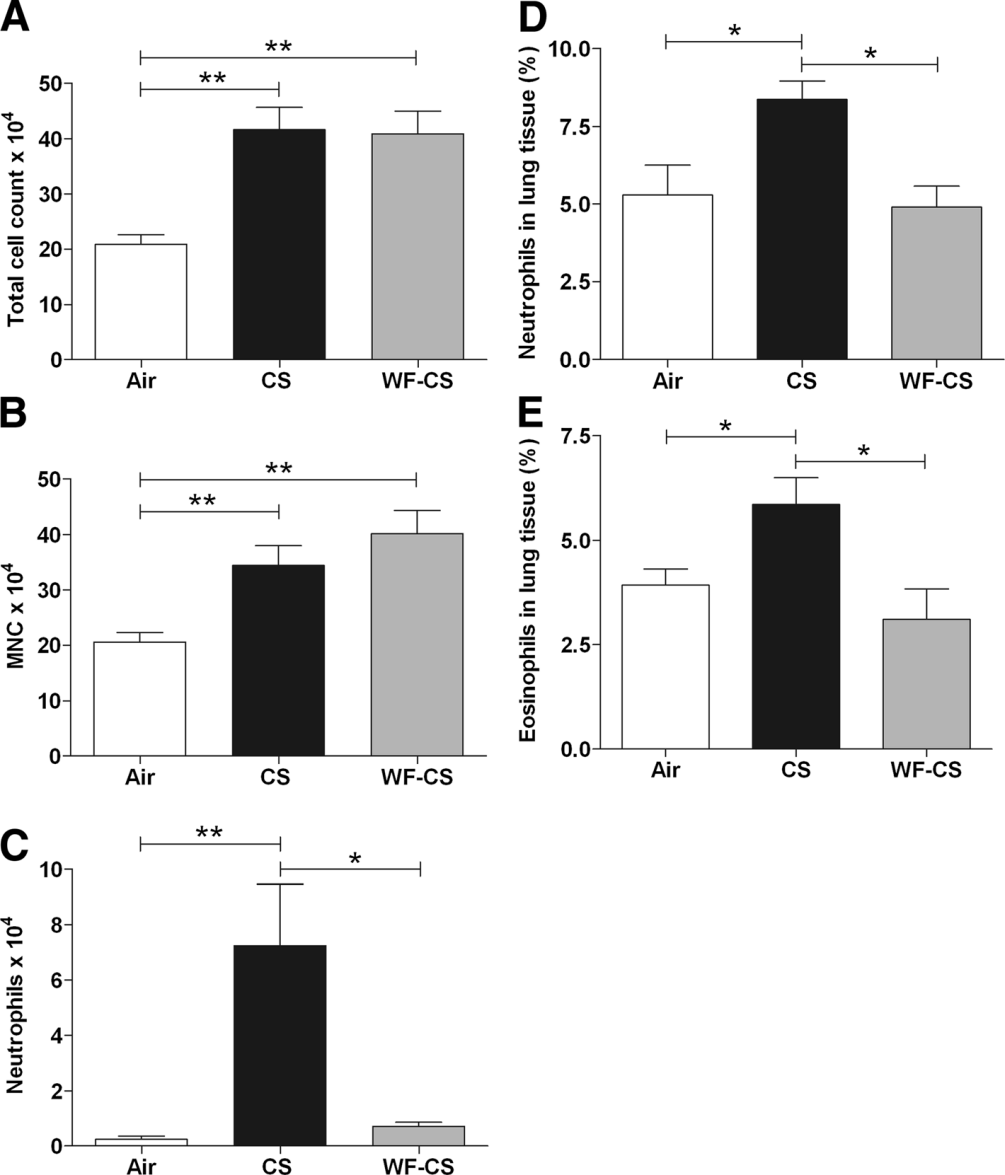
**Inflammatory cells in bronchoalveolar lavage fluids and lung tissue of mice after cigarette smoke (CS), water-filtered cigarette smoke (WF-CS) and air exposure.** Absolute numbers of (**A**) total cells, (**B**) mononuclear cells (MNC), (**C**) neutrophils in bronchoalveolar lavage fluid. Volume percentages of (**D**) neutrophils and (**E**) eosinophils in lung tissue. Data are expressed as mean values ± SEM and are referred to 7 mice per group. * *P* < 0.05, ** *P* < 0.01; significantly different from the air control group.

**Figure 4:**
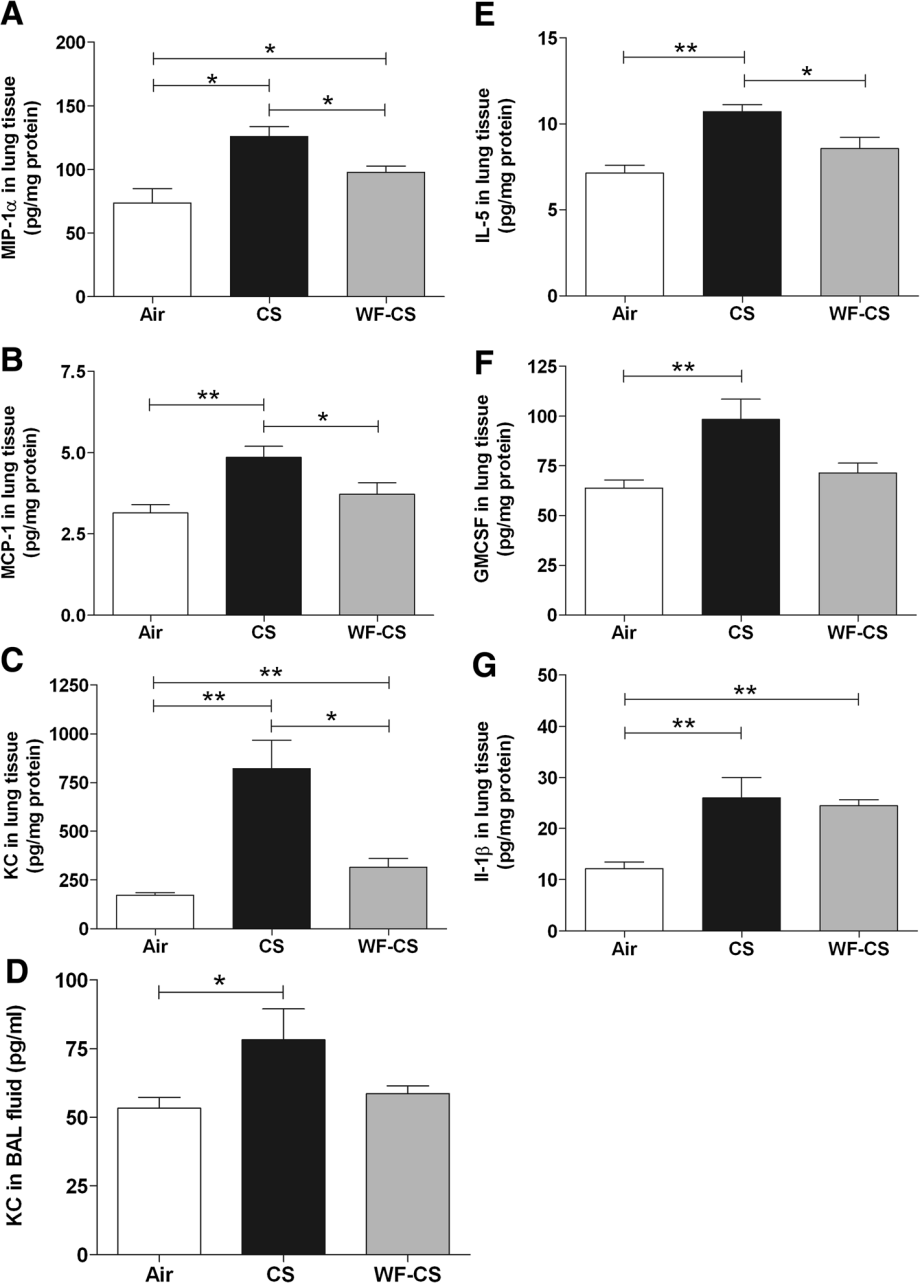
**Inflammatory cytokine levels in bronchoalveolar lavage fluid (BALF) and lung tissue homogenates of mice after cigarette smoke (CS), water-filtered cigarette smoke (WF-CS) and air exposure.** Concentration of (**A**) MIP-1α, (**B**) MCP-1, (**C**) KC expressed as pg/mg protein in lung tissue homogenates. Levels of (**D**) KC expressed as pg/ml in BALF. Concentration of (**E**) IL-5, (**F**) GM-CSF and (**G**) IL-1β expressed as pg/mg protein in lung tissue homogenates. Data are expressed as mean values ± SEM and are referred to 7 mice per group. * *P* < 0.05, ** *P* < 0.01; significantly different from the air control group.

**Figure 5:**
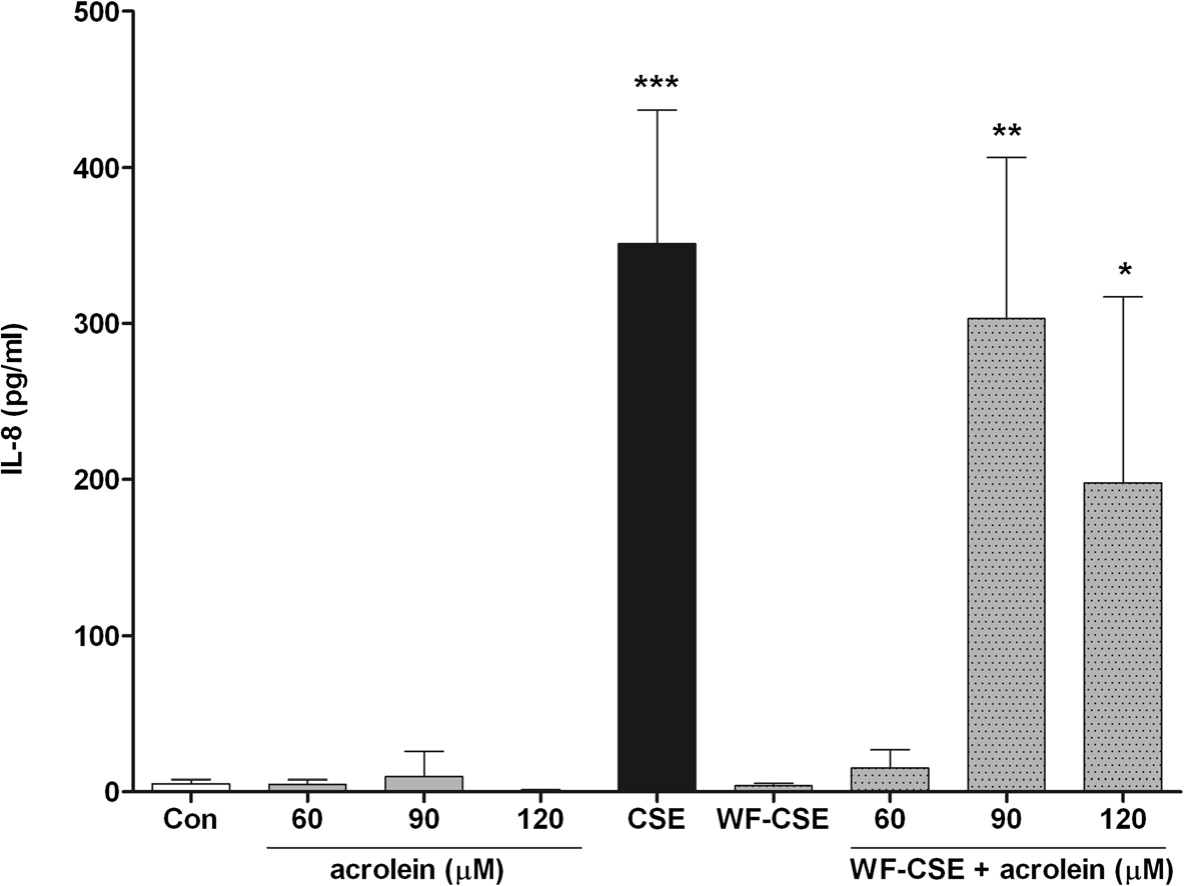
**Effect of acrolein in cigarette smoke extract (CSE) on IL-8 production in Beas-2b cells.** Production of IL-8 by Beas-2b was measured after 24 h stimulation. Data are expressed as mean values ± SEM and are referred to 3 experiments. * *P* < 0.05, ** *P* < 0.01, *** *P* < 0.0001; significantly different from the control group.

Calculations were performed using Prism 4 for Windows (GraphPad Software, San Diego, USA). Comparison between different experimental groups were performed with Student’s *t*-test (Figure 1A), Two-way ANOVA (Figure 2A), nonparametric Mann–Whitney test (Figures 3 and 4) and Dunnet’s multiple comparison test (Figure 5). *P* < 0.05 was considered significant. The Spearman's rank correlation coefficient was used to study correlations between chemokine concentrations and cellular populations of BALF. Results are presented as mean (± SEM) unless otherwise mentioned.

## Results

### Filtering CS through water slightly reduces total particle matters

Comparative smoke analyses of total particulate matter (TPM) in CS and WF-CS by trapping particles on glass fiber filters showed that filtering mainstream CS through water significantly (CS; 20.78 ± 0.59 mg, WF-CS; 16.38 ± 0.36 mg) reduces TPM (Figure 1A). GC-MS analysis showed only minor differences between CS and WF-CS (Figure 1B). The most striking differences can be traced back to volatile organic compounds (e.g. acetone (component 1), diacetyl (component 2) and methyl-ethyl ketone (component 3)).

### Filtering CS through water strongly decreases levels of aldehydes

In the present study, we were interested to selectively deplete aldehydes from CS in order to determine their contribution to CS-induced airway inflammation. In Figure 2A we demonstrate that filtering of CS through water strongly decreases (CS; 271.1 ± 41.5 μM, WF-CS; 58.5 ± 8.2 μM) the levels of total aldehydes. Differential analysis of aldehydes in CS and WF-CS using LC-UV-MS confirmed that water is able to retain aldehydes (Figure 2B). Eleven out of thirteen different aldehydes were identified in CS by mass spectrometry (Table 1). Acrolein, furancarboxaldehyde and crotonaldehyde, amongst others, were substantially reduced in intensity upon implementing the water filter.

**Table 1:** **Differential analysis of aldehydes in cigarette smoke** (**CS**) **and water**-**filtered cigarette smoke** (**WF**-**CS**) **using LC**-**UV**-**MS**

**Peak #**	**m/z**	Compound (*)	Fold change (no water/water)***
1	209,1	Formaldehyde	
2	223,1	Acetaldehyde	1,291
3	265	2,3-butanedione (isomer 1)	19,046
4	265	2,3-butanedione (isomer 2)	6,112
5	235	Acrolein	
6	275	Furancarboxaldehyde	13,087
		(isomer 1)	
7	275	Unknown (**)	42,125
8	237	Propionaldehyde	1,264
9	279	Unknown (**)	15,463
10	275	Furancarboxaldehyde	20,136
		(isomer 1)	
11	249,1	Crotonaldehyde	22,281
12	249,1	Butyraldehyde	0,748
13	265	(iso)valeraldehyde	1,48

(*) Detected as DNPH-derivatives.

(**) Unidentified aldehyde or ketone.

(***) Fold change of detected aldehydes in CS and WF-CS. Calculations were made on MS data to discriminate co-eluting species and to allow clear peak integration.

### Water filtering abrogates CS-induced lung neutrophilia and eosinophilia in mice

In order to investigate the neutrophilic airway inflammation, mice were exposed for 5 days to CS, WF-CS and air using whole body exposure. The numbers of leukocytes in BALF and lung tissue were analyzed 6 hrs after the last exposure. In Figure 3A and B we demonstrate that the total number of leukocytes (Air; 20.84 ± 1.75 × 10^4^ cells, CS; 41.63 ± 4.08 × 10^4^ cells, WF-CS; 40.87 ± 4.14 × 10^4^ cells) and the number of mononuclear cells (Air; 20.57 ± 1.73 × 10^4^ cells, CS; 34.40 ± 3.59 × 10^4^ cells, WF-CS; 40.17 ± 4.22 × 10^4^ cells) were significantly increased in BALF of mice exposed to CS and WF-CS compared to air control. Interestingly, the number of neutrophils in the BALF (Figure 3C, Air; 0.24 ± 0.12 × 10^4^ cells, CS; 7.24 ± 2.22 × 10^4^ cells, WF-CS; 0.71 ± 0.14 × 10^4^ cells) and percentages of neutrophils and eosinophils in the lung tissue (Figure 3D, Air; 5.29 ± 0.96%, CS; 8.36 ± 0.59%, WF-CS; 4.90 ± 0.68% and Figure 3E, Air; 3.93 ± 0.38%, CS; 5.86 ± 0.63%, WF-CS; 3.10 ± 0.73%) were significantly increased in the CS-exposed but not in WF-CS-exposed mice as compared to air control.

### WF-CS reduces proinflammatory cytokine levels in BALF and lung tissue homogenate

Selective chemotactic factors for the recruitment of mononuclear cells and neutrophils are macrophage inflammatory protein-1 alpha (MIP-1α), monocyte chemotactic protein-1 (MCP-1) and murine equivalent of human IL-8 (KC). In Figure 4 we demonstrate that MIP-1α (Figure 4A, Air; 73.6 ± 11.3 pg/mg protein, CS; 125.9 ± 8.0 pg/mg protein, WF-CS; 97.7 ± 4.9 pg/mg protein), MCP-1 (Figure 4B, Air; 3.14 ± 0.26 pg/mg protein, CS; 4.86 ± 0.34 pg/mg protein, WF-CS; 3.71 ± 0.36 pg/mg protein) and KC (Figure 4C, Air; 172.3 ± 13.8 pg/mg protein, CS; 821.4 ± 145.1 pg/mg protein, WF-CS; 314.4 ± 45.9 pg/mg protein) were all significantly increased in the lung tissue of CS-exposed mice when compared to air control mice. KC and MIP-1α levels were also slightly increased in the WF-CS-exposed mice when compared to control mice. Besides a significant increase in BALF KC levels in mice exposed to CS (Figure 4D, Air; 53.3 ± 10.5 pg/ml, CS; 78.1 ± 19 pg/ml, WF-CS; 58.7 ± 7.5 pg/ml) we observed no significant changes in BALF for all other cytokines tested above (data not shown). Additionally, significant correlations were obtained comparing mononuclear cells in the BALF with the concentrations of MCP-1 (Spearman r = 0.66, P<0.0093) and MIP-1a (Spearman r = 0.80, P<0.0006) in mice exposed to CS but not in mice exposed to WF-CS.

Lung tissue IL-5 levels were increased in the group of CS exposed mice when compared to controls (Figure 4E, Air; 7.14 ± 0.46 pg/mg protein, CS; 10.71 ± 0.42 pg/mg protein, WF-CS; 8.57 ± 0.65 pg/mg protein). Furthermore, lung tissue GM-CSF levels increased significantly in the CS mice and, to a lesser extent, in the WF-CS mice (Figure 4F; Air; 63.71 ± 4.17 pg/mg protein, CS; 98.29 ± 10.04 pg/mg protein, WF-CS; 71.43 ± 4.90 pg/mg protein) when compared to air control mice. Remarkably, IL-1β levels increased significantly in both groups of smoking mice (Figure 4G, Air; 12.14 ± 1.30 pg/mg protein, CS; 26.00 ± 3.99 pg/mg protein, WF-CS; 24.43 ± 1.19 pg/mg protein) when compared to air control mice.

### CS induced release of IL-8 in Beas-2b cells is aldehyde dependent

To further determine the role of aldehydes in proinflammatory responses upon CS exposure, we evaluated the effect of CSE on the production of the proinflammatory cytokine IL-8 by the bronchial epithelial cell line Beas-2b. Total concentration of aldehydes in CSE was equal to 214.2 ± 20.9 μM (data not shown). Prestimulation of Beas-2b cells with 100% CSE for 30 min showed a significant increase in the production of IL-8 within the next 24 hrs (Figure 5). Furthermore, stimulation of Beas-2b cells with WF-CSE for 30 min did not induce the production of IL-8 within the next 24 hrs. Remarkably, prestimulation with acrolein alone at concentrations from 60 to 120 μM did not stimulate IL-8 production by Beas-2b cells. However, repletion of WF-CSE with 90 and 120 μM acrolein restored the IL-8 production by Beas-2b cells.

## Discussion

In this study, we tested whether aldehydes present in gas phase CS are critical for the production of proinflammatory cytokines and cellular inflammation in the lung of mice. First, we demonstrated that WF-CS strongly reduces level of aldehydes in CS, but only has a minor effect on the concentration of other components in CS. This methodological tool was used to investigate the role of aldehydes present in CS in a mouse model of subchronic CS-induced airway inflammation [23]. We showed that the CS-induced increase in proinflammatory cytokines (e.g. MIP-1α, MCP-1, KC, IL-5 and GM-CSF) in the lungs of mice was strongly reduced when WF-CS was used. More importantly, CS-induced lung neutrophilia and eosinophilia was completely abrogated when WF-CS was used, whereas the increase in mononuclear cells remains intact. The critical proinflammatory role of aldehydes was further investigated in the human bronchial epithelial cell line Beas-2b. CSE but not WF-CSE induced the proinflammatory cytokine and neutrophil chemoattractant IL-8 in Beas-2b cells. Although acrolein itself did not induce IL-8 production in Beas-2b cells, repletion of WF-CSE with acrolein fully restored IL8 production to the level of CSE.

Aldehydes have been established as a major group of reactive components present in the gas phase of CS [12]. In line herewith, we demonstrated by LC-UV-MS that CS contains both saturated (e.g. formaldehyde, acetaldehyde, 2,3-butanedione, propionaldehyde, butyraldehyde and valeraldehyde) and unsaturated aldehydes (e.g. acrolein, furancarboxaldehyde and crotonaldehyde). These components are highly soluble in water due to high dipole moments of their carbonyl groups. Furthermore, the solubility of aldehydes in water is depending on the length of the carbon chain. Long chain aldehydes are less soluble in water compared to short chain aldehydes [24]. Our data confirmed that aldehydes from the gas phase of CS were soluble in water but we did not find major differences between the solubility of short- and long chain, saturated or unsaturated aldehydes. This might be explained by the fact that the solubility of gas phase aldehydes in water is different then that from liquid aldehydes. Comparison of GC-MS chromatograms obtained from CS and WF-CS showed difference between volatile organic compounds (e.g. acetone, diacetyl and methyl-ethyl ketone). Furthermore, there were differences in TPM between CS and WF-CS but the major differences were found by LC-UV-MS in the quantity of aldehydes. We can not exclude that these compounds contribute to the different effects of CS and WF-CS in our studies.

In this study we used a mouse model of subchronic CS-induced neutrophilic airway inflammation consisting of twice daily whole body exposure of mice for five days [23,25]. During the inflammatory response different mediators like MIP-1α, MCP-1, KC, IL-5, GM-CSF and IL-1β are secreted into the lung. Furthermore, neutrophils, eosinophils and monocytes are recruited from the blood into the lung tissue. We demonstrated that mice exposed to WF-CS were significantly protected against acute CS-induced increases of proinflammatory cytokines except for the cytokine IL-1β and were protected against recruitment of lung neutrophils and eosinophils except for macrophages in the airways of mice. Although the role of IL-1β in COPD is still unknown, this cytokine is mainly produced during acute inflammation by activated macrophages as a pro-form, which is proteolytically processed in the NLRP3 inflammasome to its active form by caspase-1 [26]. We assume that TPM inside CS and WF-CS is responsible for the activation and infiltration of pulmonary monocytes and macrophages. Pulmonary macrophages are important for clearance of inhaled TPM [27]. In vitro studies in human pulmonary macrophages have shown that TPM alone is able to activate macrophages which produce a broad spectrum of inflammatory cytokines including IL-1β [28]. In line herewith, a study by Tamagawa and colleagues demonstrated that lung inflammation in rabbits induced by TPM was characterized by increased numbers of activated pulmonary macrophages but not neutrophils [29]. In this study we demonstrate that recruitment of mononuclear cells in the BALF was significant correlated with the concentrations of MCP-1 and MIP-1a in mice exposed to CS but we did not observe any relation between levels of MCP-1 as well as MIP-1α and recruitment of mononuclear cells in mice exposed to WF-CS.

In COPD, the nature of the inflammatory process in response to CS is primarily neutrophilic. Neutrophil accumulation is a dynamic process that consists of the recruitment of neutrophils from the bloodstream and its clearance from the lungs as a result of apoptosis. Finkelstein et al. [19] demonstrated that unsaturated aldehydes generated during lipid peroxidation or CS itself are able to suppress apoptosis of neutrophils. This inhibitory effect was associated with a direct cysteine modification of the active-side cysteine residue within caspase-3 and an overall inhibition of caspase-3 activity. In addition, a variety of pro-inflammatory cytokines have also been demonstrated to delay neutrophil apoptosis in vitro; these include e.g. granulocyte macrophage-colony stimulating factor (GM-CSF) and IL-8, both of which have been shown to be increased in the airways of subjects with COPD and in our mice exposed to CS [1].

Bronchiolar epithelial cells play an important role in the recruitment of neutrophils to the site of inflammation. Bronchiolar epithelial cells are part of the innate immune system and are the first line of defense against noxious particles and gasses. Aldehydes present in CS directly leads to epithelial cell injury through protein/lipid modification and depletion of glutathione [17,18,20-22]. Dysregulated airway epithelial function appears to trigger chemoattractant mediators like IL-8 which recruit neutrophils to the site of injury [4,18]. In this study we demonstrated that depletion of aldehydes in WF-CSE significantly reduced IL-8 production in Beas-2b which could be restored by the aldehyde acrolein. This outcome is consistent with Mio T. *et all* who showed that depletion of CSE from volatile components (among them are α,β-unsaturated aldehydes) abolished the stimulatory effect of CSE on IL-8 production in human bronchial epithelial cells [18]. The decrease of IL-8 protein synthesis seems to be mediated by abrogation of the ERK1/2 and p38 MAPK pathway, one of the pivotal pathways which control IL-8 production [4]. The present data show that the levels of neutrophil chemoattractant KC and GM-CSF as well as the numbers of neutrophils in the BALF and lung tissue were significantly increased in the CS-exposed mice. Depletion of aldehydes from CS completely abrogated the infiltration of neutrophils in the BALF and lung tissue which may be explained by preventing the activation of the ERK1/2 and p38 MAPK pathway resulting in decreased levels of KC and GM-CSF in the airways.

The presence of airway eosinophilia, elevated eosinophil cationic protein levels as well as increased levels of eotaxin-1 have been reported in sputum and BALF of patients with COPD and during acute COPD exacerbations [30-32]. In these subjects, sputum eosinophilia correlated with both neutrophilic inflammation and indices of airway obstruction. Interestingly, we also observed an aldehyde dependent increase of eosinophils in the lung tissue of CS-exposed mice. Remarkably, BALF eosinophilia could not be observed in our study. This might be explained by the observation that eotaxin-2, an important factor for the recruitment of eosinophils into BALF, is first expressed in lung tissue and is only detected in the BALF of BALB/c mice at later time-points [33]. Furthermore, recruitment of eosinophils and increased eosinophil survival is mediated by GM-CSF and IL-5. These cytokines may originate from airway epithelial cells, type 2 innate lymphoid cells, T-cells and macrophages at the site of inflammation [34-36]. In line, we observed increased levels of GM-CSF and IL-5 in lung tissue of mice exposed to CS. Depletion of aldehydes from CS completely abrogated levels of GM-CSF and IL-5 as well as the recruitment of eosinophils in the lungs of mice.

## Conclusions

Our study reveals that aldehydes present in CS play an important role in the production of proinflammatory cytokines and recruitment of inflammatory cells into the airways. Our results clearly show that airway inflammation occurs both in CS-exposed mice as well as in WF-CS-exposed mice. However, specific airway and lung neutrophilia and eosinophilia, as well as corresponding pro-inflammatory cytokine levels, are aldehyde dependent in our model. Macrophage infiltration and the production of IL-1β however seems to be aldehyde independent and might be driven by other substances in smoke e.g. TPM. Future studies are needed to elucidate the precise reaction pathway of aldehydes leading to recruitment of inflammatory cells into the lungs of COPD patients.

## Abbreviations

COPD: Chronic obstructive pulmonary disease; CS: Cigarette smoke; WF-CS: Water filtered CS; CSE: CS extract; WF-CSE: WF-CS extract; MCP-1: Monocyte chemotactic protein-1; MIP-1α: Macrophage inflammatory protein-1α; GM-CSF: Granulocyte-macrophage colony stimulating factor; IL: Interleukin; KC: Murine keratinocyte-derived chemokine; TNF-α: Tumor necrosis factor-alfa; NF-κB: Nuclear factor kappa-light-chain-enhancer of activated B cells; TPM: Total particulate matter; GC-MS: Gas chromatography–mass spectrometry; DNPH: 2,4-dinitrophenylhydrazine; HPLC: High-performance liquid chromatography; LC-UV-MS: Liquid chromatography-ultraviolet spectrophotometry-mass spectrometry; SIM: Selected ion monitoring; BALF: Bronchoalveolar lavage; BAL fluid: Bronchoalveolar lavage fluid; NLRP3: NACHT, LRR and PYD domains-containing protein 3; ERK: Extracellular-signal-regulated kinase.

## Competing interests

The authors declare that they have no competing interests.

## Authors’ contributions

MT participated in the design of the study, experimentation, performed the statistical analysis and drafted the manuscript. DS participated in the design of the study and analysis of the data. HB and RG carried out the mouse experiments. RG performed the cell differentiation in the BALF and tissue. DR carried out the multiplex ELISA measurements in BALF and tissue and performed statistical analysis. LJ and KS carried out the GC-MS, HPLC and LC-UV-MS measurements and statistical analysis. DS and AO contributed to the conception of the study, participated in the interpretation of the data and critically revised the manuscript. All authors read and approved the final manuscript.
